# Potential Impacts of *Interleukin-17A* Promoter Polymorphisms on the *EGFR* Mutation Status and Progression of Non-Small Cell Lung Cancer in Taiwan

**DOI:** 10.3390/genes12030427

**Published:** 2021-03-17

**Authors:** Kai-Ling Lee, Tsung-Ching Lai, Yao-Chen Wang, Pei-Chun Shih, Yi-Chieh Yang, Thomas Chang-Yao Tsao, Tu-Chen Liu, Yu-Ching Wen, Lun-Ching Chang, Shun-Fa Yang, Ming-Hsien Chien

**Affiliations:** 1 Graduate Institute of Clinical Medicine, College of Medicine, Taipei Medical University, Taipei 11031, Taiwan; d118107005@tmu.edu.tw (K.-L.L.); rafiyang@tmu.edu.tw (Y.-C.Y.); 2 Division of Pulmonary Medicine, Department of Internal Medicine, Taipei Medical University Hospital, Taipei 110301, Taiwan; 3 Division of Pulmonary Medicine, Department of Internal Medicine, Wan Fang Hospital, Taipei Medical University, Taipei 11696, Taiwan; 109053@w.tmu.edu.tw; 4 School of Medicine, Chung Shan Medical University, Taichung 40201, Taiwan; wang5717@ms21.hinet.net (Y.-C.W.); his885889@gmail.com (T.C.-Y.T.); 5 Department of Internal Medicine, Chung Shan Medical University Hospital, Taichung 40201, Taiwan; 6 Department of Laboratory Medicine, National Taiwan University Hospital, Taipei 100, Taiwan; peichun1976@gmail.com; 7 Department of Medical Research, Tungs’ Taichung MetroHarbor Hospital, Taichung 433, Taiwan; 8 Department of Chest Medicine, Cheng-Ching General Hospital, Taichung 40764, Taiwan; liou.dj@gmail.com; 9 Department of Urology, Wan Fang Hospital, Taipei Medical University, Taipei 11696, Taiwan; s811007@yahoo.com.tw; 10 Department of Urology, School of Medicine, College of Medicine, Taipei Medical University, Taipei 11031, Taiwan; 11 Department of Mathematical Sciences, Florida Atlantic University, Boca Raton, FL 33431, USA; changl@fau.edu; 12 Institute of Medicine, Chung Shan Medical University, Taichung 40201, Taiwan; 13 Department of Medical Research, Chung Shan Medical University Hospital, Taichung 40201, Taiwan; 14 Pulmonary Research Center, Wan Fang Hospital, Taipei Medical University, Taipei 11696, Taiwan; 15 Traditional Herbal Medicine Research Center, Taipei Medical University Hospital, Taipei 110301, Taiwan; 16 TMU Research Center of Cancer Translational Medicine, Taipei Medical University, Taipei 11031, Taiwan

**Keywords:** interleukin 17A, polymorphism, epidermal growth factor receptor, mutation, lung adenocarcinoma

## Abstract

Non-small cell lung cancer (NSCLC) is a typical inflammation-associated cancer, and lung adenocarcinoma (LUAD) is the most common histopathological subtype. Epidermal growth factor receptor (EGFR) mutations are the most common driver mutations of LUAD, and they have been identified as important therapeutic targets by EGFR tyrosine kinase inhibitors. Interleukin (IL)-17A secreted by T-helper 17 lymphocytes is a proinflammatory cytokine that plays an important role in cancer pathogenesis. The present study was designed to investigate the possible associations among IL-17A genetic polymorphisms, EGFR mutation status, and the clinicopathologic development of LUAD in a Taiwanese population. Our study population consisted of 277 LUAD patients harboring the wild-type (WT) EGFR or a mutant (MT) EGFR. Four single-nucleotide polymorphisms (SNPs) of IL-17A in the peripheral blood, including rs8193036(C > T), rs8193037(G > A), rs2275913(G > A), and rs3748067(C > T) loci, were genotyped using a TaqMan allelic discrimination assay. Our results showed that none of these IL-17A SNPs were correlated with the risk of developing mutant EGFR. However, patients with a smoking habit who carried the GA genotype of IL-17A rs8193037 had a significantly lower susceptibility to EGFR mutations (adjusted odds ratio (AOR): 0.225; 95% confidence interval (CI): 0.056~0.900, *p* = 0.035). Moreover, compared to individuals carrying the CC genotype of rs8193036 at IL-17A, T-allele carriers (CT + TT) were at higher risk of developing more-advanced stages (stage III or IV; *p* = 0.020). In the WT EGFR subgroup analysis, IL-17A rs8193036 T-allele carriers had higher risks of developing an advanced tumor stage (*p* = 0.016) and lymphatic invasion (*p* = 0.049). Further analyses of clinical datasets revealed correlations of IL-17 receptor A (IL-17RA) and IL-17RC expressions with a poor prognosis of LUAD patients with a smoking history or with higher levels of tumor-infiltrating lymphocytes. In conclusion, our results suggested that two functional promoter polymorphisms of IL-17A, i.e., rs8193036 and rs8193037, were associated with the EGFR mutation status and progression in LUAD patients, indicating that these two genetic variants might act as possible markers for predicting patients’ clinical prognoses.

## 1. Introduction

Lung cancer is a highly invasive malignant tumor and is the leading cause of cancer-related mortality in the world [1]. Around 90% of patients have non-small-cell lung cancer (NSCLC), and others have small-cell lung cancer (SCLC). Lung adenocarcinoma (LUAD) comprises the greatest proportion of NSCLC cases [2]. The epidermal growth factor receptor (EGFR) is an important glycoprotein in LUAD, and EGFR mutations was shown to promote tumorigenesis. EGFR mutations in LUAD patients predict the responsiveness to EGFR tyrosine kinase inhibitor (TKI) treatment and a prolonged duration to recurrence after curative surgery [3,4,5,6].

The etiology of lung cancer is complicated and currently still incompletely understood. It was suggested that the combined effects of many genetic variants and environmental factors may contribute to cancer development [7]. Many researchers have focused on investigating immunity and inflammation in cancer development [8] and lung cancer was also demonstrated to be a typical inflammation-associated cancer [9]. Several studies suggested that genetic polymorphisms of inflammation pathway-related genes, especially cytokines and their receptors, may play roles in the susceptibility to lung cancer development [10,11].

Interleukin (IL)-17 is one of the most important members of the proinflammatory cytokine family which is composed of six members (IL-17A to IL-17F) and five receptors (IL-17RA to IL-17RE). IL-17A is the prototypical member of the IL-17 cytokine family, preferentially produced by T-helper 17 (Th17) cells, a subset of cluster of differentiation 4-positive (CD4+) T helper lymphocytes [12]. Emerging evidence revealed that IL-17A functions in an array of chronic inflammatory diseases, autoimmune disorders, and various malignancies [13,14]. High levels of IL-17A in various cancer types, including NSCLC, were reported. An increased presence of IL-17A-positive cells and high levels of IL-17A are associated with lymphatic vessel density (LVD), the smoking status, an advanced cancer stage (III/IV), and the poor survival of NSCLC patients [15,16]. Recently, IL-17A was reported to induce activation of wild-type (WT) and mutant (MT) EGFR and its downstream extracellular signal-regulated kinase 5 (ERK5), thereby promoting wound healing and skin tumorigenesis. The extracellular domain of the EGFR is not required for this action, but the kinase domain is necessary, and the EGFR TKI, gefitinib, can abolish IL-17A-induced EGFR transactivation [17].

Single nucleotide polymorphisms (SNPs) are common types of genetic variations in the genome which can alter the function and expression of coding genes associated with risks of developing certain cancers. Previous studies disclosed that IL-17A SNPs can affect the risk of cancer development and progression through influencing IL-17A expression in various malignancies including NSCLC [18,19,20]. Previously, cancer-related SNPs and somatic mutations were independently studied. However, recent research works in cancer development have explored the relationship between genetic variants and somatic mutations. For example, telomerase reverse transcriptase (TERT) SNP at rs2736100 was shown to be correlated with susceptibility to EGFR mutations in NSCLC [21]. Moreover, some cancer-related SNPs at five loci, namely, TERT, butyrophilin-like 2, TP63, bromodomain PHD finger transcription factor, and high-mobility group box protein 1 were found to have significant impacts on EGFR-mutated LUAD [22,23]. All the above illustrate a possible close interplay between SNPs and somatic mutations in oncogenesis. Accordingly, we hypothesized that IL-17A SNPs may reveal a relationship with different EGFR statuses that can alter the clinicopathological features and prognoses of LUAD patients.

To date, limited research has been conducted of examining the role of IL-17A SNPs in affecting EGFR mutation susceptibility in LUAD, especially in Asian populations. Moreover, impacts of the coexistence IL-17A SNPs with different EGFR mutation statuses on the clinicopathological characteristics of LUAD remain unknown. Hence, we performed a case-control study in a Taiwanese population to investigate correlations between IL-17A SNPs and EGFR phenotypes and their influence on the clinicopathological stage, with the aim of revealing potential genetic markers that affect the EGFR mutation status and prognosis of LUAD patients.

## 2. Materials and Methods

### 2.1. Subject Selection and Specimens

In total, 277 LUAD patients with or without an EGFR mutation were recruited from Taichung Cheng-Ching General Hospital (Taichung, Taiwan) from 2012 to 2015. Demographic characteristics of each LUAD patient, including age, gender, smoking habit, clinical stage, TNM status, and cell differentiation, were obtained from their medical records. The clinical staging of each patient was determined by the seventh edition of the TNM staging system of the American Joint Committee on Cancer Staging Manual. Informed consent was obtained from all participants, and the study protocol was approved by the Institutional Review Board of Cheng-Ching General Hospital (no. HP120009; 22 September 2012).

### 2.2. Genomic DNA Extraction from Tumor Tissues and EGFR Sequencing

Genomic DNA from the paraffin-embedded tumor tissues was extracted using either the automated QIAsymphony extraction system with a QIAsymphony DNA kit (Qiagen, Valencia, CA, USA) or QIAmp DNA Tissue kit (Qiagen). To further classify the DNA genome into MT or WT EGFR, matrix-assisted laser desorption/ionization-time of flight mass spectrometry (MALDI-TOF MS) was used as described previously [24,25], and the results were analyzed using the MassARRAY system (Agena Bioscience, San Diego, CA, USA).

### 2.3. Selection of IL-17A SNPs and Genotyping of IL-17A SNPs from Peripheral Blood

Four SNPs across IL-17A, viz., rs8193036(C > T), rs8193037(G > A), rs2275913(G > A), and rs3748067(C > T) loci, located in the promoter region or 3′ UTR were examined in this study due to their considerable associations with various malignancies, including NSCLC, and chronic inflammatory respiratory diseases, such as chronic obstructive pulmonary disease, in previous studies [18,19,20,26,27]. Peripheral blood samples from LUAD patients were collected in EDTA-containing tubes for genomic DNA extraction using the QIAamp DNA Blood Mini kit (Qiagen). Allelic discrimination of the four IL-17A SNPs was surveyed by the TaqMan SNP Genotyping Assay which utilized the ABI StepOnePlus™ Real-Time PCR System (Applied Biosystems, Foster City, CA, USA). The final collected data were further analyzed with ABI StepOnePlus™ Software vers. 2.3.

### 2.4. Cell Culture

A549 LUAD cells were purchased from and validated by the American Type Culture Collection (ATCC, Manassas, VA, USA) and cultured in RPMI 1640 medium supplemented with 10% FBS and 1% penicillin–streptomycin–glutamine (Life Technologies, Gaithersburg, MD, USA). During our studies, A549 cells were incubated at 37 °C in a humidified 5% CO_2_ atmosphere.

### 2.5. Western Blot Analysis

The detail processes of the protein lysates preparation and Western blot were according to our previous study [28]. Primary antibodies used in the Western blot analysis are as follows. The anti-IL-17A antibody (sc-374218) was purchased from Santa Cruz Biotechnology (Santa Cruz, CA, USA). Antibodies specific for His (H1029) and β-actin were all purchased from Sigma-Aldrich (St. Louis, MO, USA).

### 2.6. DNA Construction and Transient Transfection

The his-IL-17A plasmid was kindly provided by Dr. C. C. Chen and Dr. H. P. Kuo (Department of Medicine, Taipei Medical University). To overexpress IL-17A, semiconfluent cultures of A549 cells in a 6-mm^2^ Petri dish were transfected with 5 μg of an empty or expression vector (pENTER) using Invitrogen Lipofectamine 2000 Transfection Reagent. After incubation for 24 h, the cells were analyzed for the expressions of IL-17A by a Western blot analysis to confirm the overexpressed status.

### 2.7. Cell Proliferation and Colony Formation Assays

IL-17A-overexpressed A549 cells (5 × 10^3^) were seeded in 96-well plates with complete media for 24, 48, and 72 h, and a cell proliferation assay was performed by CCK8 assay (Sigma-Aldrich) according to the manufacturer’s instructions. For the assessment of colony-forming ability, IL-17A-overexpressed cells (5 × 10^2^) were seeded in six-well culture plates under standard conditions for 7 days and the medium was replaced every 3 days. At the end of experiment, the cells were stained with crystal violet, and colonies were manually counted using ImageJ free software (National Institutes of Health, Bethesda, MD, USA).

### 2.8. Outcome Analysis in Kaplan Meier (KM) Plotter

The KM plotter is a publicly available database (http://kmplot.com/analysis/index.php?p=background, accessed on 16 March 2021) which was used to study the relationship between the expression of IL-17RA or IL-17RC gene and LUAD patient clinical outcomes. We evaluated the prognostic value of IL-17RA or IL-17RC expression by Affymetrix GeneChip or RNA sequencing (RNAseq) in LUAD. The patients were divided into two groups including high and low expression of IL-17RA or IL-17RC, based on the best cut-off values of gene expression (smallest *p* value). The best cut-off values were determined by algorithms embedded in KM plotter. The overall survival (OS) KM plots are presented with the hazard ratio (HR), the 95% confidence interval (CI), and the log-rank *p*-value. We also used KM plotter to compare the predictive value of IL-17RA or IL-17RC gene in LUAD patients with or without tobacco smoking, high levels of tumor infiltrating lymphocytes, and high mutational burden (TMB). Cases in Affymetrix GeneChip were enrolled from multiple Gene Expression Omnibus (GEO) datasets and The Cancer Genome Atlas (TCGA). Cases in RNAseq were mainly recruited from TCGA. The number of genes with mutation was computed for each sample. In this, multiple mutations in a single gene count as one. Then, the median number (across all samples) was determined, and samples with more mutation were defined as high TMB.

### 2.9. Statistical Analysis

SAS statistical software, vers. 9.4 (SAS Institute, Cary, NC, USA) or Statistical Package for Social Science software, vers. 16 (SPSS, Chicago, IL, USA) was used for all statistical analyses. Differences in demographic variables between the WT and MT EGFR groups were calculated by a Mann–Whitney U-test. A multiple logistic regression was used to obtain adjusted odds ratios (AORs) with 95% CIs for different IL-17A SNP distributions between WT and MT EGFR populations after controlling for other variables. To investigate associations between clinicopathological characteristics and EGFR mutation status with different IL-17A SNPs, a multiple logistic regression was used. In in vitro study, data comparisons were performed with the Student’s *t*-test when two groups were compared. A *p* value of < 0.05 signified a statistically significant difference.

## 3. Results

### 3.1. General Characteristics of LUAD Patients with the WT or MT EGFR

The characteristics of the study population are shown in Table 1. Of 277 total LUAD patients enrolled in the study, 152 were females and 125 were males, and their ages ranged 52.11~79.27 years. Of the study population, 109 had the WT EGFR and 168 had an MT EGFR. The number of patients who had never smoked (*n* = 179) in this cohort was about 2 times that of patients with a smoking history (179 vs. 98). Compared to the WT EGFR group, the MT EGFR group had a higher female ratio (64.3% vs. 35.7%, *p* < 0.001), more nonsmokers (77.4% vs. 22.6%, *p* < 0.001), and a higher rate of greater differentiation (94.0% vs. 6.0%, *p* < 0.001). The tumor stage and tumor/node/metastasis (TNM) statuses of the WT EGFR and MT EGFR groups did not significantly differ. Overall, the demographic characteristics of our recruited LUAD subjects with an MT EGFR were consistent with those previously reported for Asian LUAD patients [29].

### 3.2. Distribution of IL-17A Candidate SNPs (rs8193036, rs8193037, rs2275913, and rs3748067) of LUAD Patients and Their Associations with the EGFR Mutation Status

To examine possible associations of IL-17A SNPs with the risk of developing EGFR mutations, the genotypes and allelic frequencies of four SNPs (rs8193036 (promoter region, C > T), rs2275913 (promoter region, -197G > A), rs8193037 (promoter region, G > A), and rs3748067 (3′ untranslated region, C > T)) were first investigated in all LUAD patients harboring the WT or MT EGFR. After adjusting for age, gender, and cigarette smoking status, no association was observed between IL-17A SNPs and the EGFR mutation status in this Taiwanese LUAD population, as calculated by both the dominant model and codominant model (*p* > 0.05) (Table S1). In addition, cigarette smoking was reported to impact the EGFR mutation rate in a Taiwanese LUAD population [30]. In the subgroup analysis stratified by subjects with or without a smoking habit, we observed that smokers with IL-17A GA heterozygotes of rs8193037 were correlated with lower frequencies of developing EGFR mutations (AOR: 0.225, 95% CI = 0.056~0.900, *p* = 0.035) (Table 2). In contrast, there was no significant difference in the correlations of IL-17 SNPs with WT or MT EGFR status in non-smoking LUAD patients (Table S2)

### 3.3. Correlations between Polymorphic Genotypes of IL-17A and Clinicopathological Characteristics of LUAD Patients with the WT or MT EGFR

We next evaluated the impacts of these IL-17A SNPs on clinicopathological characteristics of LUAD patients, including the cancer stage, primary tumor size, lymph node involvement, distant metastasis, and cell differentiation, and the results are shown in Table 3. We divided overall LUAD patients into two subgroups: patients who had homozygous reference alleles and those who had at least one polymorphic allele. We observed that the IL-17A rs8193036 dominant model was significantly correlated with a more-advanced stage (III or IV) of LUAD patients (AOR: 1.976; 95% CI: 1.114~3.506, *p* = 0.020) (Table 3), while no associations were found for IL-17A rs8193037, rs2275913, or rs3748067 with clinicopathologic characteristics of LUAD patients (data not shown). We further divided our recruited LUAD patients into WT and MT EGFR groups and observed that IL-17A rs8193036 T allele carriers (CT or TT) with the WT EGFR had enhanced risks of developing advanced-stage tumors (stage III or IV; AOR: 4.175; 95% CI: 1.312~13.285, *p* = 0.016) and lymph node metastasis (AOR: 2.839; 95% CI: 1.005~8.018, *p* = 0.049) (Table 4). In contrast, no significant association between rs8193036 and the clinicopathologic status was observed in LUAD patients with an MT EGFR (Table S3).

### 3.4. Effects of IL-17A on Growth of LUAD Cells Harboring WT EGFR and MT KRAS

**Figure 1:**
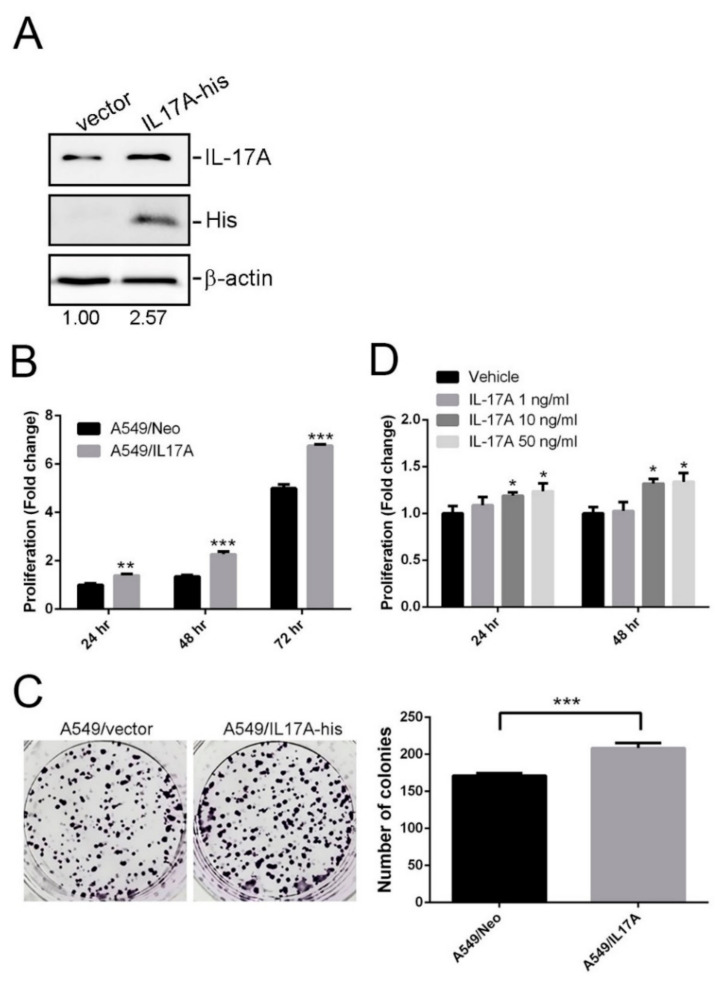
IL-17A promotes proliferative and colony forming abilities of A549 lung adenocarcinoma (LUAD) cells harboring wild-type (WT) EGFR and a mutant (MT) KRAS. (**A**) Western blot analysis of IL-17A expressions in A549 cells after transfecting an IL-17A-expressing vector (IL-17A-His). (**B**,**D**) Proliferation rates of IL-17A-overexpressed (**B**) or IL-17A recombinant-treated (**D**) A549 cells were measured by performing CCK8 assays. viability. ** *p* < 0.01, *** *p* < 0.001, compared to the control (A549/Neo) group. * *p* < 0.05, compared to vehicle treatment group. (**C**) Effects of IL-17A on the long-term growth (7 days) of A549 cells were evaluated using a colony formation assay. Left panel: Representative photomicrographs. Right panel: Data are presented as the mean ± SD of three independent experiments. *** *p* < 0.001, compared to the control group.

EGFR and KRAS are known as the most commonly mutated gene in NSCLC and the mutations of these two genes are thought to be mutually exclusive. Herein, we used A549 LUAD cell line harboring the WT EGFR and MT KRAS as a cell model. We overexpressed His-tagged IL-17A in A549 cells (Figure 1A) and evaluated their tumorigenic abilities. Compared to vector control-transfected cells, IL-17A-overexpressing cells showed significant increases in the proliferative (Figure 1B) and colony forming (Figure 1C) abilities. Moreover, treatment of A549 cells with IL-17A recombinant also induced proliferation of cells in a concentration-dependent manner (Figure 1D).

### 3.5. Prognostic Significance of IL-17 Receptors in LUAD Patients with Different Smoking or EGFR Statuses

**Figure 2:**
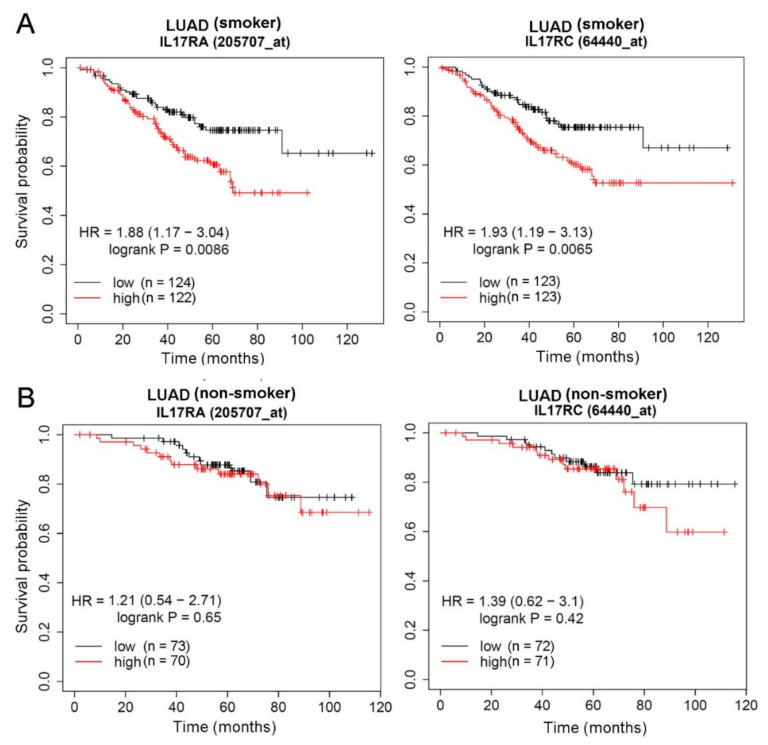
Prognosis of interleukin-17 receptor A (IL-17RA) and IL-17RC in lung adenocarcinoma (LUAD) patients with different smoking statuses. (**A**) Correlations of IL-17RA and IL-17RC expression levels with the overall survival (OS) rate in LUAD patients with a smoking history. (**B**) Correlations of IL-17RA and IL-17RC expression levels with the OS rate in LUAD patients without a smoking history. The Affymetrix ID is valid: 205707_at (IL-17RA) or 64440_at (IL-17RC). Gene expressions were dichotomized into high and low values using the median as a cutoff. HR, hazard ratio.

**Figure 3:**
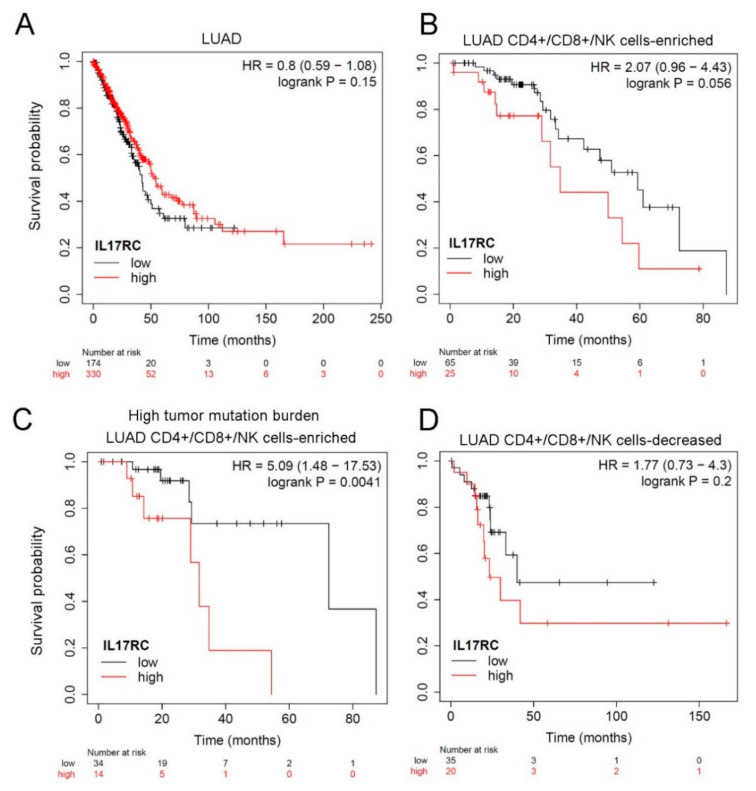
Prognostic value of the expression of interleukin 17 receptor C (IL-17RC) in patients with lung adenocarcinoma (LUAD) stratified by immune cell infiltration and tumor mutation burden statuses. Kaplan-Meier overall survival curves are plotted based on (**A**) all LUAD patients (*n*  =  504), (**B**) cluster of differentiation 4-positive (CD4+)/CD8+/natural killer (NK) cell-enriched LUAD patients (*n* = 90), (**C**) CD4+/CD8+/NK cell-enriched LUAD patients with a high tumor mutation burden (*n* = 48), and (**D**) CD4+/CD8+/NK cell-decreased LUAD patients (*n* = 55).

IL-17A and IL-17F exist either as homodimers or as heterodimers, and all forms of the cytokine induce signals through a dimeric IL-17RA and IL-17RC receptor complex. Both IL-17RA and IL-17RC play essential roles in disease progression including cancer [31]. In addition, interactions between IL-17A SNPs and tobacco smoking were reported to be correlated with the risk of lung cancer [26]. Considering potential effects of IL-17A SNPs on IL-17A expression levels, we further clarified the clinical significance of its receptors, IL-17RA and IL-17RC, as expressed by LUAD tissues. Correlations between IL-17RA or IL-17RC expression and OS in LUAD patients were analyzed using the KM plotter. All tested LUAD patients from the GEO and TCGA databases were divided into smokers and non-smokers, and we found that significant correlations of IL-17RA and IL-17RC expressions with OS times were only observed in the smoking population (Figure 2A), not in the non-smoking population (Figure 2B). From the RNAseq analysis, IL-17RC showed no prognostic effect in LUAD patients (Figure 3A). As far as we know, IL-17A is produced by CD4+ T cells, CD8+ T cells, and natural killer (NK) T cells [14]. We therefore restricted our analysis to LUAD patients with tissue-enriched CD4+, CD8+, and NK T cells, and found those with higher IL-17RC levels had a trend of being associated with a poorer prognosis (*p* = 0.056) (Figure 3B), and this correlation was more striking in the population with a high tumor mutation burden (*p* = 0.0041) (Figure 3C). In contrast, LUAD tissues containing decreased CD4+, CD8+, and NK T cells showed that IL-17RC levels were not correlated with OS of LUAD patients (Figure 3D). Taken together, the above clinical data suggest that expression of the IL-17A-IL-17RA/TL-17RC axis may modulate the progression of LUAD, especially in patients with high levels of tumor infiltrating lymphocytes and a high tumor mutation burden.

## 4. Discussion

The demographic and basic characteristics of our LUAD cohort were similar to previous Asian cohorts, in which females, nonsmokers, and those with better cell differentiation were more frequently observed among LUAD patients with an MT EGFR compared to the WT EGFR group. Nonsmoking was correlated with the development of LUAD, while more nonsmokers had EGFR mutations [32]. This is the first study to examine associations of IL-17A SNPs with the EGFR mutation status and clinicopathologic characteristics in a Taiwanese LUAD population. We provide a novel finding that LUAD patients with a smoking history harboring IL-17A rs8193037 GA heterozygotes had a significantly lower incidence of developing an EGFR mutation. Moreover, LUAD patients harboring the WT EGFR and a T allele rs8193036 polymorphism had higher risks of developing advanced-stage tumors and lymph node metastasis.

Smoking is an important environmental factor that is associated with the initiation of lung cancer by tobacco carcinogens and with the promotion of tumor development through the induction of inflammation [8,33]. Smoking is related to IL-17A upregulation in NSCLC [16] through affecting promoter polymorphisms of the IL-17A gene that enhance the susceptibility to lung cancer [19,20,26]. KRAS and EGFR mutations in NSCLC are thought to be mutually exclusive. KRAS-mutant tumors, which are associated with smoking and inflammation, display significantly higher levels of IL-17A compared to EGFR-mutant tumors [34]. NSCLC patients harboring the rs8193037A allele were reported to produce significantly more IL-17A than those with the GG genotype [20]. We hypothesize that LUAD patients with the rs8193037 GA genotype who smoke exhibit higher expression level of IL-17A and prevalence of the KRAS mutation and a lower prevalence of EGFR mutation. Our results showed that a statistically significant association occurred between IL-17A rs8193037 GA genotypes and lower MT EGFR risks in a specific group of LUAD patients who were smokers, but the KRAS mutation status and IL-17A level in this subgroup should be further checked in the future. Stratification analyses from a previous study showed that the rs8193037A allele had a significantly higher prevalence in LUAD patients than in those with squamous cell carcinoma [20]. Taken together, our novel finding suggests that GA alleles of rs8193037 might be a determinative genetic variation for the presence of EGFR mutations in LUAD patients who are smokers, and therefore may affect their clinical course and prognosis.

We further investigated whether SNPs of IL-17A affected the clinicopathologic characteristics of LUAD patients. In earlier studies, elevated IL-17A expression was observed in individuals with the rs8193036 T allele [35]. From in vitro, in vivo, and clinical studies, IL-17A was reported to induce proliferation, the inhibition of apoptosis, and the migration of NSCLC cell lines and tumor growth and metastasis in an NSCLC xenograft model; and it was correlated with an advanced stage of NSCLC (III/IV) or a poor OS in NSCLC patients [36]. Our data revealed that rs8193036 T-allele carriers were associated with significantly more-advanced stages (III + IV) among LUAD patients, suggesting that IL-17A rs8193036 SNPs promote tumor progression probably via upregulation of IL-17A expression. Interestingly, IL-17A overexpression was reported to enhance the growth of lung tumors carrying a KRAS mutation in mice [34]. Due to the mutations of KRAS and EGFR in NSCLC are mutually exclusive, we divided our LUAD patients into WT and MT EGFR subgroups and found that the T allele of rs8193036 was only correlated with an advanced stage and lymph node metastasis in LUAD patients harboring the WT EGFR. We suggest that IL-17A rs8193036 SNP might affect LUAD tumor progression and its clinical course in patients harboring the WT EGFR and MT KRAS. Accordingly, we further tested the effect of IL-17A on an A549 LUAD cell line harboring the WT EGFR and an MT KRAS. We found that IL-17A-overexpressing A549 cells exhibited higher proliferative and colony forming abilities compared to vector control cells. Moreover, treatment of A549 cells with IL-17A recombinant also induced proliferation of cells. The effects of IL-17A rs8193036 SNPs on IL-17A expression and progression in A549 cells are worthy to further investigate in future.

IL-17A needs to bind with its receptor complex, including IL-17RA and IL-17RC, and further activate downstream pathways to achieve its biological effects, such as promoting invasion in NSCLC cells [37]. Several clinical studies showed that IL-17RA expression was higher in various tumor tissues, including NSCLC [38]. In our clinical assessment, we observed that high levels of IL-17RA and IL-17RC were correlated with poor prognostic outcomes in LUAD patients with a smoking history, but not in nonsmokers. Moreover, IL-17A is produced by CD4+ T cells, CD8+ T cells, and NK T cells [14]. We observed that in populations with T lymphocytes-infiltrating LUAD tissues that expressed high levels of IL-17RC messenger (m)RNA had poorer prognoses, particularly in those with a high tumor mutation burden. Collectively, the above clinical data suggest that tobacco smoking, tumor-infiltrating T lymphocytes, and the tumor mutation burden may play critical roles in IL-17A-IL-17RA/IL-17RC-modulated LUAD progression.

Some limitations are recognized in the current study. First, the sample size of the current study was still not large enough and might lead to a limited statistical impact on the accuracy and precision of the results. For example, in some statistical analyses of selected subgroups (e.g., lymph node metastasis status in LUAD patients with WT EGFR), the rather small number of patients might cause that the statistical significance *p* value was practically irrelevant (*p* = 0.049). Ultimately, larger independent cohort from other medical centers are needed to further confirm the impact of IL-17A SNPs on EGFR mutation susceptibility and LUAD development. Moreover, this study is restricted to the Taiwanese population (of Asian or Chinese ethnicity), other ethnic populations is necessary to compare with and double-check our current results. Furthermore, our study cannot validate the association between genetic variants of IL-17A with expression level of IL-17A in LUAD. Therefore, the mRNA and DNA should be collected simultaneously from the same samples to further validate this issue in future work.

## 5. Conclusions

In this study, we first identified that IL-17A rs8193037 SNP was significantly associated with lower EGFR mutation risks in LUAD patients who smoked and that IL-17A rs8193036 SNP was associated with an advanced clinical course particularly in patients with the WT EGFR. These two IL-17A promoter SNPs can possibly be utilized as potential biomarkers in the absence of an MT EGFR in LUAD patients who smoke, as well as a cancer aggressiveness predictor for WT EGFR LUAD patients. Moreover, we also indicated that T lymphocyte infiltration in the tumor microenvironment is critical for IL-17A-IL-17RA/IL-17RC-modulated LUAD progression.

## Figures and Tables

**Table 1:** Clinical characteristics of lung adenocarcinoma patients with the wild type or mutation type of the epidermal growth factor receptor (EGFR).

Subject Characteristic	Total (*N* = 277)	Wild Type (*N* = 109)	Mutation Type (*N* = 168)	*p* Value
**Age, *n* (%)**				
Mean ± SD (years)	65.60 ± 13.46	65.45 ± 13.34	65.69 ± 13.58	0.885
**Gender, *n* (%)**				
Male	125 (45.1%)	65 (59.6%)	60 (35.7%)	<0.001
Female	152 (54.9%)	44 (40.4%)	108 (64.3%)	
**Cigarette smoking, *n* (%)**				
Non-smoker	179 (64.6%)	49 (45.0%)	130 (77.4%)	<0.001
Ever-smoker	98 (35.4%)	60 (55.0%)	38 (22.6%)	
**Cancer stage, *n* (%)**				
I or II	72 (26.0%)	25 (22.9%)	47 (28.0%)	0.350
III or IV	205 (74.0%)	84 (77.1%)	121 (72.0%)	
**Tumor T status, *n* (%)**				
T1 or T2	166 (59.9%)	59 (54.1%)	107 (63.7%)	0.113
T3 or T4	111 (40.1%)	50 (45.9%)	61 (36.3%)	
**Lymph node status, *n* (%)**				
Negative	81 (29.2%)	28 (25.7%)	53 (31.5%)	0.295
Positive	196 (70.8%)	81 (74.3%)	115 (68.5%)	
**Distant metastasis, *n* (%)**				
Negative	133 (48.0%)	53 (48.6%)	80 (47.6%)	0.870
Positive	144 (52.0%)	56 (51.4%)	88 (52.4%)	
**Cell differentiation, *n* (%)**				
Good/Moderate	245 (88.4%)	87 (79.8%)	158 (94.0%)	<0.001
Poor	32 (11.6%)	22 (20.2%)	10 (6.0%)	

Categorical data: *n* (%); continuous variables: mean ± standard deviation (SD). The Mann-Whitney U-test or Fisher’s exact test was used to evaluate the comparisons between the EGFR wild type and mutation type in lung adenocarcinoma patients. A *p* value < 0.05 was defined as statistically significant.

**Table 2:** Distribution frequencies of interleukin (*IL*)-*17A* genotypes of lung adenocarcinoma patients with the ever-smoking status and multiple logistic regression analysis of EGFR mutation associations.

Genotype SNP	Wild Type (*N* = 60)	Mutated Type (*N* = 38)	AOR (95% CI)	*p* Value
**rs8193036**				
CC	36 (60.0%)	19 (50.0%)	1.00	
CT	18 (30.0%)	12 (31.6%)	1.675 (0.628~4.469)	0.303
TT	6 (10.0%)	7 (18.4%)	3.125 (0.842~11.590)	0.088
CT + TT	24 (40.0%)	19 (50.0%)	2.023 (0.837~4.890)	0.117
**rs8193037**				
GG	44 (73.3%)	35 (92.1%)	1.00	
GA	15 (25.0%)	3 (7.9%)	**0.225 (0.056~0.900)**	0.035 *
AA	1 (1.7%)	0 (0.0%)	---	---
GA + AA	16 (26.7%)	3 (7.9%)	**0.222 (0.056~0.885)**	0.033 *
**rs2275913**				
GG	23 (38.3%)	8 (21.1%)	1.00	
GA	23 (38.3%)	19 (50.0%)	2.021 (0.712~5.742)	0.186
AA	14 (23.4%)	11 (28.9%)	1.523 (0.450~5.154)	0.499
GA + AA	37 (61.7%)	30 (78.9%)	1.839 (0.689~4.911)	0.224
**rs3748067**				
CC	49 (81.7%)	32 (84.2%)	1.00	
CT	10 (16.7%)	6 (15.8%)	0.811 (0.258~2.550)	0.720
TT	1 (1.6%)	0 (0.0%)	---	---
CT + TT	11 (18.3%)	6 (15.8%)	0.721 (0.234~2.223)	0.569
**rs763780**				
TT	40 (66.7%)	27 (71.1%)	1.00	
TC	15 (25.0%)	10 (26.3%)	0.941 (0.343~2.586)	0.906
CC	5 (8.3%)	1 (2.6%)	0.411 (0.042~3.999)	0.444
TC + CC	20 (33.3%)	11 (28.9%)	0.824 (0.319~2.124)	0.688

Abbreviation: SNP, single-nucleotide polymorphism. The adjusted odds ratios (AORs) with their 95% confidence intervals (CIs) were estimated by multiple logistic regression models after controlling for age and gender. * A *p* value of < 0.05 was defined as statistically significant.

**Table 3:** Clinicopathologic characteristics of lung adenocarcinoma patients stratified by polymorphic genotypes of interleukin (*IL*)-*17A* rs8193036.

Genotype SNP	CC (*N* = 153)	CT or TT (*N* = 124)	AOR (95% CI)	*p* Value
**Stage**				
I or II	48 (31.4%)	24 (19.4%)	1.00	0.020 *
III or IV	105 (68.6%)	100 (80.6%)	**1.976 (1.114~3.506)**	
**Tumor T status**				
T1 or T2	91 (59.5%)	75 (60.5%)	1.00	0.813
T3 or T4	62 (40.5%)	49 (39.5%)	0.942 (0.574~1.545)	
**Lymph node metastasis**				
Negative	49 (32.0%)	32 (25.8%)	1.00	0.249
Positive	104 (68.0%)	92 (74.2%)	1.372 (0.801~2.348)	
**Distant metastasis**				
Negative	73 (47.7%)	60 (48.4%)	1.00	0.846
Positive	80 (52.3%)	64 (51.6%)	0.953 (0.586~1.550)	
**Cell differentiation**				
Good/Moderate	131 (85.6%)	114 (91.9%)	1.00	0.122
Poor	22 (14.4%)	10 (8.1%)	0.527 (0.234~1.187)	

* *p* < 0.05 was defined as statistically significant. The AORs with their 95% CIs were estimated by multiple logistic regression models after controlling for age, gender, and the cigarette smoking status.

**Table 4:** Clinicopathologic characteristics of lung adenocarcinoma patients with wild-type epidermal growth factor receptor (EGFR), stratified by polymorphic genotypes of interleukin (*IL*)-*17A* rs8193036.

Genotype SNP	CC (*N* = 63)	CT or TT (*N* = 46)	AOR (95% CI)	*p* Value
**Stage**				
I or II	20 (31.7%)	5 (10.9%)	1.00	0.016 *
III or IV	43 (68.3%)	41 (89.1%)	**4.175 (1.312~13.285)**	
**Tumor T status**				
T1 or T2	34 (54.0%)	25 (54.3%)	1.00	0.691
T3 or T4	29 (46.0%)	21 (45.7%)	0.849 (0.377~1.908)	
**Lymph node metastasis**				
Negative	21 (33.3%)	7 (15.2%)	1.00	0.049 *
Positive	42 (66.7%)	39 (84.8%)	**2.839 (1.005~8.018)**	
**Distant metastasis**				
Negative	34 (54.0%)	19 (41.3%)	1.00	0.273
Positive	29 (46.0%)	27 (58.7%)	1.587 (0.696-3.620)	
**Cell differentiation**				
Good/Moderate	48 (76.2%)	39 (84.8%)	1.00	0.271
Poor	15 (23.8%)	7 (15.2%)	0.553 (0.192-1.590)	

* *p* < 0.05 was defined as statistically significant. The adjusted odds ratios (AORs) with their 95% confidence intervals (CIs) were estimated by multiple logistic regression models after controlling for age, gender, and the cigarette smoking status.

## Data Availability

The datasets generated for this study are available on request to the corresponding authors or in the GEO and TCGA databases.

## Supplementary Materials

The following are available online at https://www.mdpi.com/2073-4425/12/3/427/s1, Table S1: Distribution frequencies of interleukin (IL)-17A genotypes of patients with lung adenocarcinoma and multiple logistic regression analysis of epidermal growth factor receptor (EGFR) mutation associations; Table S2. Distribution frequencies of interleukin (IL)-17A genotypes of lung adenocarcinoma patients with the non-smoking status and multiple logistic regression analysis of epidermal growth factor receptor (EGFR) mutation associations; Table S3. Clinicopathologic characteristics of lung adenocarcinoma patients with MT EGFR, stratified by polymorphic genotypes of interleukin (IL)-17A rs8193036.
