# Supplementary material for: Potential Impacts of Interleukin-17A Promoter Polymorphisms on the EGFR Mutation Status and Progression of Non-Small Cell Lung Cancer in Taiwan
Source: Genes (Basel). 2021 Mar 17;12(3):427. doi: 10.3390/genes12030427 (PMC8002550; doi:10.3390/genes12030427)
Supplement: Supplementary file 1 [file genes-12-00427-s001.pdf]

Supplementary data

Table S1. Distribution frequencies of interleukin (*IL*)-*17A* genotypes of patients with lung adenocarcinoma and multiple logistic regression analysis of epidermal growth factor receptor (EGFR) mutation associations.

Genotype SNP	Wild type (*N* = 109)	Mutated type (*N* = 168)	AOR (95% CI)	*p* value
**rs8193036**				
CC	63 (57.8%)	90 (53.6%)	1.00	
CT	39 (35.8%)	63 (37.5%)	1.077 (0.619~1.875)	0.792
TT	7 (6.4%)	15 (8.9%)	2.402 (0.850~6.793)	0.098
CT+TT	46 (42.2%)	78 (46.4%)	1.241 (0.736~2.093)	0.418
**rs8193037**				
GG	83 (76.1%)	136 (81.0%)	1.00	
GA	25 (22.9%)	31 (18.5%)	0.706 (0.370~1.348)	0.292
AA	1 (1.0%)	1 (0.5%)	0.619 (0.025~15.360)	0.770
GA+AA	26 (23.9%)	32 (19.0%)	0.703 (0.372~1.329)	0.278
**rs2275913**				
GG	32 (29.4%)	43 (25.6%)	1.00	
GA	48 (44.0%)	85 (50.6%)	1.115 (0.596~2.084)	0.734
AA	29 (26.6%)	40 (23.8%)	0.857 (0.417~1.761)	0.674
GA+AA	77 (70.6%)	125 (74.4%)	1.019 (0.568~1.830)	0.949
**rs3748067**				
CC	92 (84.4%)	135 (80.4%)	1.00	
CT	16 (14.7%)	32 (19.0%)	1.383 (0.684~2.799)	0.367
TT	1 (0.9%)	1 (0.6%)	0.729 (0.042~12.769)	0.829
CT+TT	17 (15.6%)	33 (19.6%)	1.341 (0.674~2.670)	0.403

Abbreviations: SNP, single-nucleotide polymorphism. The adjusted odds ratios (AORs) with their 95% confidence intervals (CIs) were estimated by multiple logistic regression models after controlling for age, gender, and the cigarette smoking status.

Table S2. Distribution frequencies of interleukin (*IL*)-*17A* genotypes of lung adenocarcinoma patients with the non-smoking status and multiple logistic regression analysis of epidermal growth factor receptor (EGFR) mutation associations.

Genotype SNP	Wild type (*N* = 49)	Mutated type (*N* = 130)	AOR (95% CI)	*p* value
**rs8193036**				
CC	27 (55.1%)	71 (54.6%)	1.00	

CT	21 (42.9%)	51 (39.2%)	0.991 (0.493~1.993)	0.979
TT	1 (2.0%)	8 (6.2%)	2.684 (0.316~22.806)	0.366
CT+TT	22 (44.9%)	59 (45.4%)	1.079 (0.545~2.137)	0.827
**rs8193037**				
GG	39 (79.6%)	101 (77.7%)	1.00	
GA	10 (20.4%)	28 (21.5%)	1.412 (0.586~3.402)	0.442
AA	0 (0.0%)	1 (0.8%)	---	---
GA+AA	10 (20.4%)	29 (22.3%)	1.443 (0.600~3.467)	0.412
**rs2275913**				
GG	9 (18.4%)	35 (26.9%)	1.00	
GA	25 (51.0%)	66 (50.8%)	0.537 (0.207~1.391)	0.200
AA	15 (30.6%)	29 (22.3%)	0.362 (0.128~1.025)	0.056
GA+AA	40 (81.6%)	95 (73.1%)	0.468 (0.189~1.159)	0.101
**rs3748067**				
CC	43 (87.8%)	103 (79.2%)	1.00	
CT	6 (12.2%)	26 (20.0%)	1.763 (0.657~4.733)	0.260
TT	0 (0.0%)	1 (0.8%)	---	---
CT+TT	6 (12.2%)	27 (20.8%)	1.827 (0.682~4.893)	0.231
**rs763780**				
TT	33 (67.3%)	87 (66.9%)	1.00	
TC	16 (32.7%)	39 (30.0%)	0.909 (0.442~1.871)	0.796
CC	0 (0.0%)	4 (3.1%)	---	---
TC+CC	16 (32.7%)	43 (33.1%)	0.998 (0.489~2.044)	0.986

Abbreviation: SNP, single-nucleotide polymorphism. The adjusted odds ratios (AORs) with their 95% confidence intervals (CIs) were estimated by multiple logistic regression models after controlling for age and gender.

Table S3. Clinicopathologic characteristics of lung adenocarcinoma patients with MT EGFR, stratified by polymorphic genotypes of interleukin (*IL*)-*17A* rs8193036.

**Genotype SNP**	**CC (N = 90)**	**CT or TT (N = 78)**	**AOR (95% CI)**	***p* value**
**Stage**				
I or II	28 (31.1%)	19 (24.4%)	1.00	0.309
III or IV	62 (68.9%)	59 (75.6%)	1.440 (0.714~2.905)	
**Tumor T status**				
T1 or T2	57 (63.3%)	50 (64.1%)	1.00	0.854
T3 or T4	33 (36.7%)	28 (35.9%)	0.940 (0.485~1.822)	

**Lymph node metastasis**				
Negative	28 (31.1%)	25 (32.1%)	1.00	0.813
Positive	62 (68.9%)	53 (67.9%)	0.922 (0.471~1.805)	
**Distant metastasis**				
Negative	39 (43.3%)	41 (52.6%)	1.00	0.316
Positive	51 (56.7%)	37 (47.4%)	0.723 (0.384~1.362)	
**Cell differentiation**				
Good/Moderate	83 (92.2%)	75 (96.2%)	1.00	0.459
Poor	7 (7.8%)	3 (3.8%)	0.577 (0.134~2.476)	

Abbreviations: SNP, single-nucleotide polymorphism. The adjusted odds ratios (AORs) with their 95% confidence intervals (CIs) were estimated by multiple logistic regression models after controlling for age, gender, and the cigarette smoking status.
